# Lessons from Pasteur may help prevent the deadly relapse of Ebola in patients: Using contingency vaccination to avoid Ebola relapse in immune-privileged organs

**DOI:** 10.3389/fimmu.2023.1060481

**Published:** 2023-03-20

**Authors:** Wei Ye, Chuantao Ye, Jia Li, Yingfeng Lei, Fanglin Zhang

**Affiliations:** ^1^ Department of Microbiology, School of Preclinical Medicine, Airforce Medical University: Fourth Military Medical University, Xi’an, Shaanxi, China; ^2^ Department of Infectious Diseases, Tangdu Hospital, Airforce Medical University: Fourth Military Medical University, Xi’an, Shaanxi, China; ^3^ Department of Neurology, Xi’an International Medical Center Hospital, Xi’an, Shaanxi, China

**Keywords:** Ebola virus (EBOV), Ebola Virus Disease (EVD), Filovirus, neutralizing antibody (NAb), virus relapse, vaccine, contingency vaccination, immune-privileged organ

## Introduction

Ebola virus (EBOV) is one of the deadliest viruses causing severe diseases with high mortality. After the outbreak in West Africa in 2014, many specific treatment measures were tested experimentally; among these, countermeasures based on neutralizing antibodies (NAbs) have been considered one of the most practical means. However, some investigators have reported that patients with critical Ebola virus disease (EVD) could experience a deleterious recurrence of EBOV from immune-privileged organs with concomitant NAbs treatment, most prominently in the brain (1). Where EBOV is hidden and how virus relapse occurs are largely unknown. Recently, research on non-human primates (NHPs) has provided some evidence. Using this evidence and devising countermeasures to prevent the virus from recurring are a critical issue to discuss.

## Main text

EBOV persistence in cerebrospinal fluid (CSF), ocular fluid, and seminal fluid has been documented in West Africa since 2004 (2–4). A recent study by Jun Liu et al. revealed the possible origins of hidden EBOV that cause relapsed meningoencephalitis in non-human primates (NHPs) by providing detailed evidence (5). This study identified CD68^+^ macrophages as the cellular reservoir responsible for persistent EBOV brain infection but not astrocytes or neurons, which infiltrated the ventricular lumina and ependymal cell layers of brain ventricles and the choroid plexus (5). In addition, the recurrence of meningoencephalitis is due to viral multiplication in the cerebral system (5). This research partly provides a possible explanation for similar phenomena documented in patients with EVD treated with experimental NAbs (1, 6). Furthermore, such results have been suggested for other NHPs infected with hemorrhagic fever viruses treated with NAbs, such as Junin virus and Machupo virus, the causative agents of Argentine hemorrhagic fever and Bolivian hemorrhagic fever, respectively (7, 8).

Although treatment with NAbs produces positive results in EVD patients, particular attention should be paid to the possible recurrence of EBOV from immune-privileged organs in these critical patients, who generally cannot survive the infection. The proposed solutions (combination therapies of mAbs and antiviral drugs) from this research may be insufficient to eradicate the persistence of EBOV since fatal cases of EBOV infection usually have low antibody levels and an unbalanced T-cell response (9). Reconstructing the protective immune response against EBOV in these patients may help clear the virus from immune-privileged sites and prevent subsequent potential virus transmission (6).

As one of the foundations of immunology, the success of the rabies vaccine is an ideal example of contingency vaccination. The first treatment strategy is to inject attenuated rabies viruses into the victims as soon as possible. Although successful rabies treatment in the 19th century was comparatively low and lacked rigorous control, attenuated viruses from the spinal cords of rabbits that had succumbed to rabies have emerged as the first reliable treatment for rabies, with a mortality rate of nearly 100% (10). This contingency vaccination strategy has also been tested in other acute viral infections in animal models, that is, the Crimean–Congo hemorrhagic fever transcription and entry-competent virus-like particle vaccine provides complete protection against CCHFV in mice (11). Moreover, an ideal example of an emergency vaccine has been adopted as a widely accepted strategy for preventing the infection of neonates with hepatitis B virus (HBV). The universal vaccination of children starting from birth, regardless of the maternal HBV status, followed by 1 and 6 months (or 2, 4, and 6 months or so) is the cornerstone of hepatitis B eradication plans (12, 13) and has helped to substantially reduce HBV burden in China and other parts of the world. The prevalence of the hepatitis B surface antigen (HBsAg) declined from approximately 10% among children in the 1980s to <0.5% among children born after 2011 (14). Moreover, since 2012, China has implemented the national hepatitis B immunoglobulin administration program and will achieve the 2030 target set by the World Health Organization; that is, the HBsAg prevalence rate of children aged 5 years old will be less than 0.1%.

Contingency vaccination can be an alternative treatment for critical cases of EVD patients. A choice for this purpose has already been offered by successful vesicular stomatitis virus (VSV)–based EBOV vaccines (15, 16). Previous field research revealed that the efficacy of the VSV-EBOV vaccine in immediate vaccination was 100% (95% CI: 68.9–100.0, p = 0.0045) (17). However, the replication-competent VSV-based vaccine may not be the best choice for treating EVD patients because EBOV is a master to manipulate the immune system, whether the secreted glycoprotein (sGP) compensates for some NAbs or VP24 and VP35 hinder the innate immune response (18). In one study using VSV-EBOV as the post-exposure treatment for rhesus macaques infected with EBOV-Makona, as all animals in the untreated group died, 33%–67% of the animals in the treatment groups survived due to different strategies, indicating that VSV-EBOV is a potent prophylactic vaccine. The efficacy of post-exposure treatment is limited (19). Other clinical research results cannot provide reliable evidence but confirm that VSV-EBOV vaccine is well tolerated and safe for postexposure vaccination. As evidented in an observational follow-up studies of healthcare workers in contact with a late reactivation of EVD in the United Kingdom (20) or a case that experienced a needlestick while working in an Ebola treatment unit (21). However, in a case report of a relapse of systemic EVD, a patient who had been administered VSV-EBOV vaccine approximately 6 months before testing positive for EVD could not detect anti-EBOV glycoprotein (GP) Immunoglobulin G (IgG) on the first day (6). This suggests a possible failure to elicit an effective IgG response by the VSV-EBOV vaccine. Additionally, data from clinical trials in Liberia documented that approximately 20% of VSV-EBOV-vaccinated individuals did not develop positive Ebola IgG-binding titers 1 month after vaccination (22).

Meanwhile, EVD patients are in critical condition. Consequently, developing a subunit-based protein or an messenger RNA (mRNA) vaccine is a possible treatment option that can be combined with NAbs and antivirals. Moreover, since the relapse of EBOV from the immune-privileged site usually takes time, a VSV-based EBOV vaccine could be administered to recovered patients for approximately a month or other suitable time intervals. In this way, vaccines can serve as a “boost” to elicit protective immunity. Subsequent antibodies and T-cell responses could serve as an arsenal that protects patients who cannot exert a protective immune response during EBOV infection, thus preventing possible EBOV recurrence from immune-privileged organs. One critical point is the identification of EVD patients with an elapsed potential. As described in the case reports (6), upon administration, patients without signs of EBOV GP IgG or have a very low titer could serve the title of “EVD patients with elapse potential.” However, further evidence is required.

Another interesting question is that not all patients who received NAbs treatment experienced EBOV recurrence. A more comprehensive study may be required to clarify which patients are inclined to relapse. Aside from the evidence of the brains of antibody-treated non-human primate survivors (5), the NAbs titer during convalescence may be an interesting indicator. However, the virus failed to elicit effective antibodies in critical patients who normally cannot survive, and NAb treatment supplemented this deficiency. It is difficult for patients to clear away the hidden virus by themselves; in contrast, this finally leads to a relapse in immune-privileged organs. Since evidence from previous data confirmed that these kinds of patients usually fail to establish an antibody response in the first place, it is important to monitor the EBOV GP IgG titer in the follow-up survey, e.g., 1 1/2 months [approximately two times the half-life of NAbs (23)], and estimate the cut-off level for vaccine indications. After vaccine administration, it is important to monitor IgG responses in follow-up surveys. These data may eventually add to our knowledge of the nature of the antibody level that restricts viral relapse. Similar to previous studies, during the latent period of varicella-zoster virus infection, vaccination helps postpone the occurrence of herpes zoster (24).

## Conclusion

**Figure 1:**
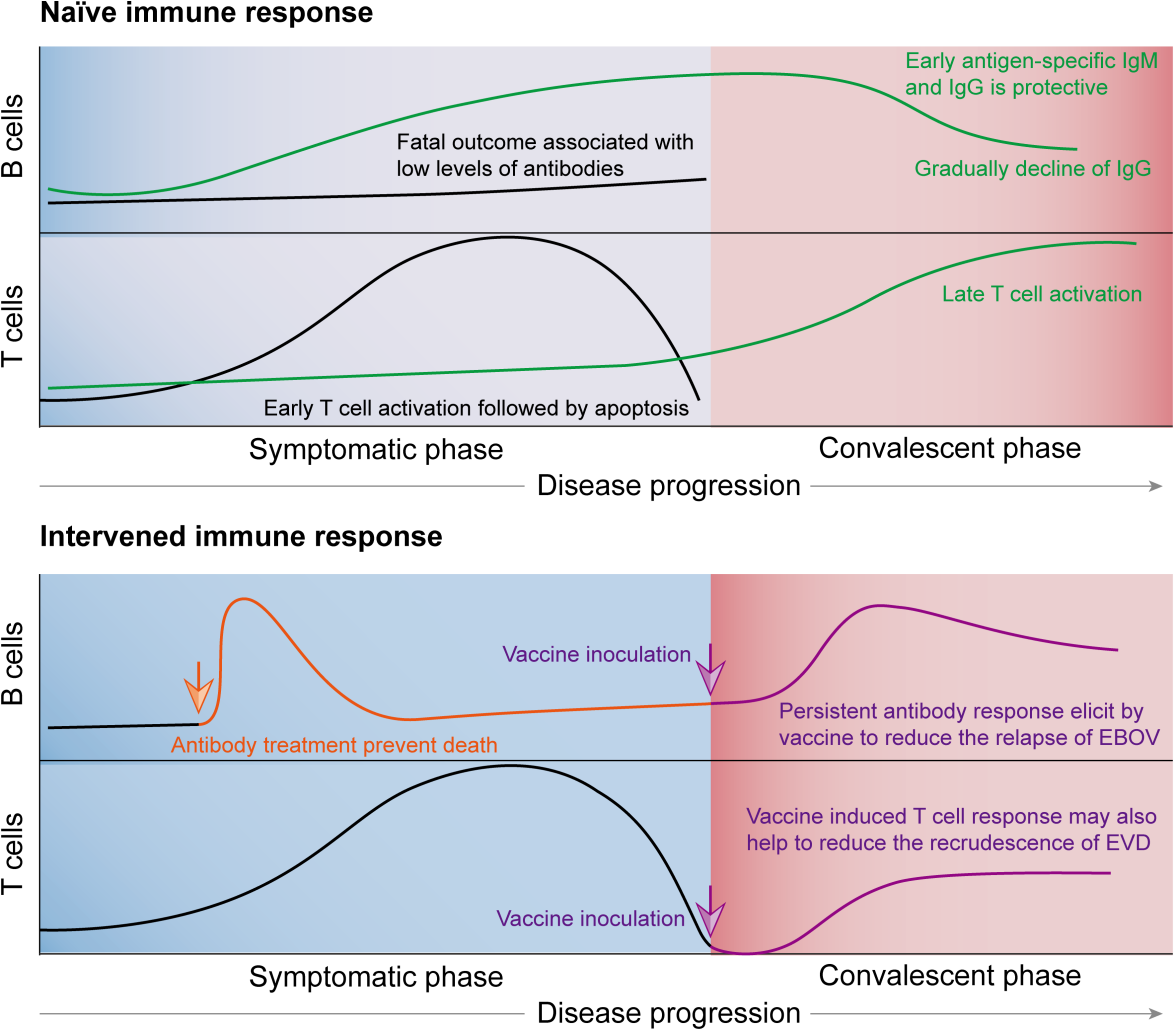
Immunological responses that correlate with fatality or survival from infection with Ebola virus (EBOV) and possible intervention strategies to treat critical cases and prevent virus persistence as well as disease recrudescence. B-cell responses, which are reflected by antibody production and early IgG and Immunoglobulin M (IgM) levels, are protective against EBOV infection. However, a fatal outcome is usually associated with low antibody response levels. Neutralizing antibody (NAb) treatment can reverse the disease outcome but cannot induce an immune response in the patients. Combining vaccine inoculation and NAb treatment could prevent fatal outcomes and elicit B- and T-cell responses in patients, which may help reduce virus persistence and disease recrudescence. T-cell responses are also crucial for surviving EBOV infection, which can be elicited by the vaccine.

To avoid a potential recurrence of EBOV in immune-sensitive organs, treatment with NAb in patients with critical EVD may be supplemented by an EBOV GP subunit vaccine or an mRNA vaccine to help reduce the possibility of relapse. NAb treatment could prevent fatal outcomes, and vaccine inoculation could elicit B-cell and T-cell responses, which may help reduce virus persistence and disease relapse (**Figure 1**). The key point is to develop a standard for evaluating the type of patients needing vaccine inoculation after convalescence. The EBOV GP IgG antibody titer upon patient administration could serve as an early warning of potential viral relapse when no obvious antibody is detected. The IgG titer at the two-time half-life of treatment with NAb after discharge may serve as the second index. However, the differences in IgG titers between fully recovered patients and those with viral relapse tendencies require further research. If one suffered EVD and had a very low EBOV GP titer upon administration and the antibody titer was still low at convalescence, it may serve as an index to receive the currently available VSV-EBOV vaccines. These strategies could also be employed for subunit-based vaccines (**Figure 1**). The antibodies and T-cell responses elicited by the EBOV vaccine could serve as an arsenal that protects patients by counteracting potential virus relapse from immune-privileged organs.

## Author contributions

YL and FZ provided administrative support and financial support. WY and JL conceived of the study and wrote the manuscript. CY prepared the figure. WY, CY, JL, YL, and FZ checked and finalized the manuscript. All authors contributed to the article and approved the submitted version.
